# MicroRNA-146: Biomarker and Mediator of Cardiovascular Disease

**DOI:** 10.1155/2022/7767598

**Published:** 2022-10-11

**Authors:** Fatemeh Sadat Mahdavi, Shayan Mardi, Sareh Mohammadi, Sarina Ansari, Somayeh Yaslianifard, Parviz Fallah, Sayed-Hamidreza Mozhgani

**Affiliations:** ^1^Student Research Committee, School of Medicine, Alborz University of Medical Sciences, Karaj, Iran; ^2^Cardiovascular Research Center, Alborz University of Medical Sciences, Karaj, Iran; ^3^Department of Microbiology, School of Medicine, Alborz University of Medical Sciences, Karaj, Iran; ^4^Department of Laboratory Science, School of Allied Medicine, Alborz University of Medical Sciences, Karaj, Iran; ^5^Noncommunicable Disease Research Center, Alborz University of Medical Sciences, Karaj, Iran

## Abstract

Cardiovascular diseases (CVDs) are the prime cause of morbidity and mortality worldwide. Although noticeable progress has been made in the diagnosis, prognosis, and treatment, there is still a critical demand for new diagnostic biomarkers and novel therapeutic interventions to reduce this disease incidence. Many investigations have been conducted on the regulatory effects of microRNAs in cardiovascular diseases. miRNA circulating serum level changes are correlated with several CVDs. In addition, there is growing evidence representing the potential role of miRNAs as diagnostic biomarkers or potential therapeutic targets for CVD. Preliminary studies identified the prominent role of miR-146 in host defense, innate immunity, and different immunological diseases by regulating cytokine production and innate immunity modification in bacterial infections. However, more recently, it was also associated with CVD development. miR-146 has received much attention, with positive results in most studies. Research demonstrated the crucial role of this molecule in the pathogenesis of cardiac disease and related mechanisms. As a result, many potential applications of miR-146 are expected. In this paper, we provide an overview of recent studies highlighting the role of miR-146 in CVD, focusing on CAD (coronary artery disease), cardiomyopathy, and MI (myocardial infarction) in particular and discussing its current scientific state, and use a prognostic biomarker as a therapeutic agent for cardiovascular diseases.

## 1. Introduction

Despite significant advances and improvements in preventive care, diagnostic tools, and treatments, cardiovascular diseases (CVDs) are still a significant cause of mortality and morbidity. They have imposed a significant burden on the health care system [1]. CVD consists of various disorders associated with cardiomyocytes, conduction system of the heart, coronary artery diseases (CAD), and congenital disabilities [2]. As a subtype of CAD, myocardial infarction is the necrosis of the myocardium, secondary to the prolonged lack of oxygen supply [3–5].

The occurrence and progression of CVD usually result from many risk factors, including genetic, epigenetic, and environmental factors. Eight WHO's targeted risk factors for reduction by 2025 are smoking, hypertension, diabetes, dyslipidemia, obesity, diet, alcohol, and sedentary lifestyle [6, 7]. According to the high mortality and disability rate of CVD, better knowledge of risk factors would probably help us quickly detect those with an increased risk of CVD development and improve patient outcomes and survival. In attempts to diagnose and treat CVD early, noncoding RNAs (ncRNAs) have recently attracted more attention [8, 9].

The role of miR-146 in host defense, innate immunity, and immunological diseases has been mentioned by various researchers. It links the innate immune to oncogenic transformation and is involved in inflammation, innate immunity, and cancer. For example, downregulation of miR-146b in lung cancer [10], increased expression of miR-146a/b in metastatic human breast cancer cells [11], and mitochondrial functions regulation have been reported earlier.

**Figure 1:**
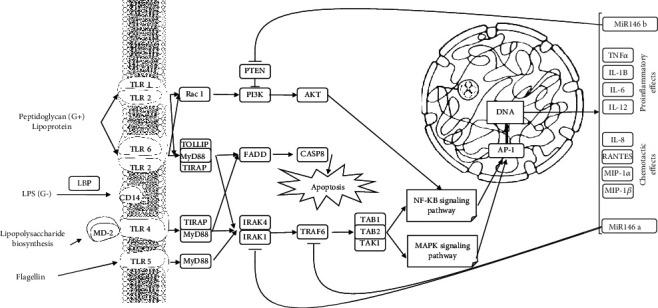
By identifying inflammatory molecules, a cascade of intermediate molecules is created, and depending on the type of activated receptor, the cell may become apoptotic or produce proinflammatory and chemotactic molecules. Inflammatory molecules activate both the MAPK signaling and NF-*κ*B signaling pathways, eventually increasing the production of inflammatory and chemotactic interleukins by affecting DNA. As shown, miR-146a and miR-146b exhibit their anti-inflammatory properties in relatively different pathways.

miR-146 plays a central role in many inflammatory processes [12]. Studies showed that in vitro treatment of miR-146a may induce the expression of proinflammatory factors such as TNF-*α*, NF-*κ*B p65, and MCP-1, which are essential transcription factors in atherosclerosis [13]. Palomer et al. showed critical functions in miR-146a in myocardial dysfunction and progression of HF. miR-146a suppresses MMP-9 by affecting c-Fos and decreasing the c-Fos/AP-1 pathway [14]. As shown in Figure 1, through its inhibitory effect on TLR4, miR-146 decreases AP-1 in the cell nucleus, diminishes cytokine production, and suppresses inflammation. In most cardiac diseases, inflammation leads to the death of cardiomyocytes and reduced cardiac contraction capacity [15]. In this condition, the tissue level of miR-146 is increased to suppress the inflammation through negative autoregulation. Various research were conducted according to these mechanisms to determine the diagnostic capacity of these biomarkers.

A study by Oerlemans et al. on the serum level of miR-146 in ACS patients showed that the serum level of this biomarker had significantly increased in non-ST segment elevation MI (NSTEMI) and unstable angina compared to non-ACS patients. Moreover, the serum level of miR-146 is higher in NSTEMI patients than in unstable angina patients [16]. Furthermore, Arroyo et al.'s study showed that the miRNA was effective on ACS in AF (atrial fibrillation) through neutrophil extracellular trap formation [17].

As it is evident, pharmacological inhibition of TNF-*α* promotes the function of myocardium cells during HF [18]. Studies reported the elevation of miR-146a transcript levels in the ventricular tissue of transgenic mice, with specific TNF-*α* overexpression in the myocardium and a human origin cardiomyocyte cell line (AC16) exposed to TNF-*α*. Target genes of miR-146a were remarkably reduced after miR-146a overexpression or TNF-*α* treatment [14]. Also, in vivo studies using a lentivirus expressing miR-146a (LmiR-146a) have shown that miR-146a is essential in preventing sepsis-induced NF-*κ*B signaling and the generation of inflammatory cytokines as well as the inhibition of IRAK and TRAF6 expression in the myocardium. Therefore, it leads to heart dysfunction secondary to sepsis [19], indicating the role of miR-146a as a novel therapeutic tool in inflammatory-associated cardiac disorders [14]. However, studies have not proven the diagnostic value of this marker [20].

Numerous studies have been conducted on miR-146 genotypes and their relationship with different diseases [21–23]. For example, Chen et al. showed an enormous increase in MI and other CADs risk by the CC genotype [24]. In contrast, further research demonstrates the association of miR-146a rs2910164 C>G genotype and low ACS risk [25]. These results depend highly on the study population, and studying in new communities can lead to different outcomes. A study by Kim et al. on hypertensive patients and the GG genotype control group demonstrated a significant association of miR-146a C>G genotype distributions with this disease [25]. Another polymorphism of this miRNA is rs2431697. Studies on CAD patients have shown that the wild type of rs2910164 and mutant type of rs2431697 are more frequent [26].

The association of ischemic stroke and mentioned polymorphisms has also been discussed separately. For example, Zhu et al. stated that the risk of ischemic stroke might increase by miR-149 T>C and miR-146a C>G polymorphisms, which might be mainly associated with an increased risk of large artery atherosclerosis stroke [27]. Zha et al. examined the allele frequencies and stated a significant association between the genotype and allele frequency of the miR-146a gene loci rs2910164 G/C and rs57095329 A/G. Also, they suggest that genotype GG of rs2910164 G/C and allele A of rs57095329 A/G are risk factors for coronary artery lesions [28].

No previous published literature has brought together the role of miR-146 on each type of CVD. In the current review, the most recent studies have been discussed, highlighting the role of miR-146 in CVD, focusing on CAD, cardiomyopathy, and MI in particular, and providing an overview of its current scientific state and use as a prognostic biomarker as well as a therapeutic agent for cardiovascular diseases. Therefore, we provide a comprehensive conclusion of the potential role of miR-146 and its relevance to different types of CVD to fill the gaps in previous studies.

In order to perform a comparative study on the effect of miR-146 on heart diseases, PubMed, the Institute for Scientific Information (ISI), Scopus, and EMBASE were searched using the following keywords:

))))))hsa-mir-146) OR miR-146, human) OR microRNA-146, human) OR hsa-mir-146b) OR hsa-mir-146a) AND ((((Heart failure) OR Cardiomyopathy) OR Myocardial infarction) OR Coronary artery diseases).

Articles were screened by title, abstract, and, eventually, the main text. Cross-sectional studies reporting descriptive data in English were included. The retrieval literature publication date was before Jan 24, 2022. The analysis did not include review articles, opinion pieces, or letters that did not include original data. The following information was extracted from each study: the authors, year, type of disease, microRNA subtype, clinical appliance, study design, study size, and source tissue. Finally, a total of 43 eligible articles were selected for further consideration.

## 2. Acute Myocardial Infarction

Although the acute myocardial infarction (AMI) diagnosis is made with an overview of the patient's history, symptoms, signs, ECG, and physical examination, cardiac biomarkers are still an essential diagnostic factor in AMI cases [29]. Troponin and its two isoforms, I and T, are widely used as specific lab tests to diagnose the disease. Research has shown that, despite this test's high specificity for myocardial injury, troponin isoforms are not highly sensitive. Also, troponin measurements in patients with STEMI are not of immediate value, and the treatment plan should still be based on a rapid troponin check, the patient's clinical symptoms, and ECG.

Studies suggest new promising biomarkers for AMI diagnosis, one of which is miRNAs [30] (Table 1). Although miR-146 plays a significant role in cardiac diseases, studies on the diagnostic value of this marker have not been promising. Xue et al. showed that circulating miR-146a can be used as biomarkers for AMI diagnosis [31]. In contrast, Corsten et al. stated that the detectable release of microRNA-208b and microRNA-499 has been seen in cardiac damage, but no significant elevation in plasma levels of miR-146 has been seen in AMI patients [20]. Also, the results of a study by Bukauskas et al. showed that although plasma levels of miR-146a-5p in STEMI patients were 4.048-fold lower than the control group, the AUC of this microRNA is less than 0.8 and is classified as a fair predictor [32].

The different results of the above studies are probably due to different methods. In the study of Xue et al., blood samples were taken from all patients in the first 4 hours, including 14 patients with STEMI and 17 with NSTEMI.

Treatment of AMI is based on two general pillars, reducing the load on the heart and restoring blood flow to the infarcted area. Despite the positive results of emergency reperfusion, this leads to the spread of inflammatory molecules throughout the heart muscle, apoptosis of myocardial cells, and thus an increase in the size of the infarct location. Nowadays, there are several approaches to controlling MI-induced I/R injury, including inhibiting leukocyte accumulation, complement inhibition (e.g., pexelizumab), and inhibition of mPTP. Unfortunately, none of the available methods can completely prevent I/R injury [33]. In 2015, Liu et al. showed that cells exposed to inflammation attempted to reduce neutrophil activity and suppress inflammation by increasing the secretion of miR-146a. The results of this study identify miR-146a as a prognostic and therapeutic target in myocardial I/R injury [34].

On the other hand, Shu et al. showed that Troxerutin could significantly control myocardial ischemia or reperfusion injury by inhibiting miR-146a-5p [35]. Due to study limitations and the fact that all studies have been limited to animal and cellular research, the need for more extensive research in clinical settings is strongly felt. Considering the role of miR-146a in the liver and renal I/R injury and the results of the mentioned articles, the prognostic and therapeutic use of this microRNA in managing this condition will probably be adequate.

A critical cause of death due to AMI is various complications such as left ventricular remodeling (LVR) or ventricular rupture. Studies have shown that the main mechanisms involved in causing these complications are immune pathways such as TLR and NF-*κ*B-mediated pathways, and due to the regulatory role of miR-146 on these pathways (Figure 1), different studies have been done in this regard. For example, Zhao et al. reported that miR-146b mediated vascular inflammation and apoptosis in MI patients [36].

The most critical steps in managing left ventricular remodeling (LVR) patients are determining prognosis and preventive measures by blood volume management and administering angiotensin-converting enzyme (ACE) inhibitors and vasodilators such as NO to prevent cardiovascular events such as cardiogenic shock [37]. So far, several factors have been suggested for determining prognosis, including C-reactive protein (CRP), creatinine kinase MB (CK-MB), troponin I plasma levels, eGFR, and LV ejection fraction [38], but these factors are not accurate enough; and new prognostic factors are required. Liu et al. in 2015 studied prognostic factors for LVR and showed that the miR-146a assessment for five days following MI could significantly predict LVR development (AUC = 0.818) [39]. Unfortunately, extensive studies have not been conducted in this area, and further clinical studies are needed (Table 2).

Ventricular rupture (VR) is one of the most acute cardiac complications requiring rapid surgical measures to prevent the patient's death. Therefore, predicting this event is very important. Various research have been done on the role of miR-146a on VR. For example, a study by Zidar et al. on tissue specimens from 50 patients who expired due to MI showed that measurement of serum level of miR-146a could be used in 2-7 days post MI as a prognostic factor for VR incidence in MI patients [40]. Research on the role of this microRNA in VR is minimal, and conclusions need to be studied. Nevertheless, it can be expected that AMI patients with higher levels of miR-146 are more likely to develop MI complications than other patients.

## 3. Myocarditis

Myocarditis is defined as myocardial inflammation associated with cardiac dysfunction, which may be due to viral, bacterial infections, or the body's autoimmune reaction. Despite various diagnostic modalities, the definite diagnosis still depends on endomyocardial biopsy, and the detection of inflammatory infiltrates in specimens according to the Dallas criteria.

Due to the difficulties in diagnosing viral myocarditis (VM), diagnostic factors have been considered. Different studies investigate the role of miR-146 in this disease; for example, cellular research conducted by Chen et al. reports that miR-146b upregulation is associated with VM by targeting ITCH. They also noted that overexpression of this miRNA is responsible for cytokine and chemokine excessive secretion, which enhances myocardial inflammation by weakening the NF-*κ*B pathway feedback signaling. Furthermore, specific immunosuppressive agents may control the inflammatory response [41]. In return, studies on the serum level of this miRNA have shown no significant elevation of this factor in VM patients compared to the control group, which probably reduces its use as a diagnostic factor [20].

Another cause of myocarditis is sepsis. Sepsis is a life-threatening organ dysfunction caused by the host's dysregulated immune response to infection [42]. Considering the role of miR-146a in immune processes, especially TLR4-mediated pathways, XIE et al. examined the changes in this miRNA by injecting bacterial LPS into 60 healthy mice. This study showed that miR-146a might inhibit the TLR4/NF-*κ*B signaling pathway through negative feedback mechanisms, thereby reducing the complications of sepsis-induced cardiomyopathy [43]. Therefore, inhibition of this miRNA can be considered a promising therapeutic approach. However, similar to the results seen in VM, the diagnostic value of this microRNA in other types of myocarditis is also not significant. For example, results of the study by Besler et al. demonstrated no significant difference concerning miR-146b levels between sepsis-induced cardiomyopathies patients and controls [44]. Also, Gumus et al. showed that the serum levels of miR-146a were similar between the acute rheumatic myocarditis (ARM) patient and control groups [45].

Due to the minimal results, mir146a could be used as a therapeutic target in various types of myocarditis. However, the preliminary results of the above studies show that the diagnostic use of this factor is not very promising due to the deficient serum level of this microRNA in patients with myocarditis. Further studies are needed to lighten whether these miRNAs might be helpful as therapeutic targets.

## 4. Cardiomyopathy

A heterogeneous group of myocardial diseases associated with electromechanical dysfunctions is classified as cardiomyopathies. These patients usually exhibit inappropriate ventricular hypertrophy or dilatation and are due to a variety of causes that frequently are genetic [46]. Diagnosis of cardiomyopathy can be made with various diagnostic techniques, including chest X-ray, treadmill stress test, ECG, cardiac catheterization, echocardiogram, and blood tests. Blood factors such as B-type natriuretic peptide (BNP) are used in cardiomyopathy diagnosis. New studies point to the diagnostic potential of miR-146 in multiple causes of cardiomyopathy.

Type 2 diabetes (T2D) is prevalent with various cardiac complications. Due to the progressive nature and cardiac complications of T2D, early diagnosis and treatment of diabetic cardiomyopathy (DC) are essential. Studies have demonstrated that the plasma level of miR-146 is associated with cardiac complications of diabetes, including DC [47]. Feng et al. showed that miR-146a is reduced in endothelial cells exposed to high glucose, which leads to the activation of NF-*κ*B, thus, other inflammatory molecules. The protective effect of miR-146a on functional and structural alterations in DC through preventing inflammatory changes [48] (Table 3).

Nevertheless, an increase in miR-146a in T2D patients has been reported by some studies. For example, Alipoor et al. suggested that higher levels of miR-146a, especially rs2910164 polymorphism, might increase the risk of T2D and its cardiac complications [49]. The apparent difference in the above results is probably due to the different roles of this microRNA in different cells. The study by Feng et al. clearly shows that endothelial cells experience a decrease in miR-146 cellular levels due to exposure to high glucose levels, resulting in intracellular inflammatory processes. Nevertheless, the role of miR-146 in diabetes is not limited to endothelial cells. Studies have shown that the deregulation of microRNAs in peripheral blood mononuclear cells (PBMC) plays a more critical role in the pathogenesis of the disease [50]. Probably what increases the risk of T2D in people with higher plasma levels of miR-146 (or different polymorphisms) is the inflammatory processes involved in PBMCs, not endothelial cells.

Despite the different results of the studies, the role of miR-146 in the pathogenesis of DC is undeniable, and most patients with DC show abnormal (and mostly high) amounts of this microRNA, but no valid studies have been performed on the diagnostic value of miR-146 for DC.

**Figure 2:**
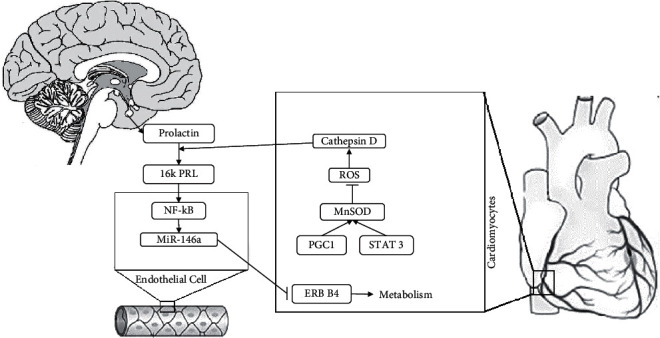
Pregnancy leads to volume overload physiologically, increasing the preload. In PPCM, cardiomyocytes become inflamed due to overwork and produce MnSOD. By inhibiting ROS, cathepsin D increases and enters the bloodstream. On the other hand, during pregnancy, a lot of prolactin is present in the bloodstream, which breaks into 16K PRL by cathepsin D. 16K PRL also affects endothelial cells and leads to the production of miR-146a. Eventually, this molecule disrupts the metabolism of heart cells by affecting cardiomyocytes and inhibiting ErbB-4.

Early diagnosis of peripartum cardiomyopathy (PPCM) is vital due to the relatively high prevalence (about 1 in 2000 cases) and the need for prompt treatment. Studies have cited various pathophysiological models for the disease, including increased cardiac susceptibility to myocarditis in pregnancy, cross-reactivity of antiuterine antibodies to the heart muscle, or oxidative stress due to prolactin breakdown [51, 52]. As stated by Halkein et al., the absence of STAT3 or PGC1 in cardiomyocytes leads to the decreased expression of the ROS scavenger MnSOD in these cells [53]. Decreased MnSOD levels accumulate ROS and increase and activate Cathepsin D, which is secreted from cardiomyocytes into the interstitial space. Cathepsin D cleaves PRL to generate an antiangiogenic 16-kDa fragment, 16K PRL. 16K PRL stimulates endothelial cells to activate NF-*κ*B through an unknown mechanism that increases miR-146a levels. miR-146a decreases NRAS levels and inhibits angiogenesis. It also transits to cardiomyocytes in exosomes to reduce ERBB4 levels, resulting in a slower cardiac metabolism (Figure 2). Also, Szczerba et al. showed that in the third trimester of physiological pregnancy, the serum level of miR-146a increases by 70%, which is probably related to its cardiac protective effects against volume overload [54]. This increase led to further inhibition of ERBB4 in PPCM patients and decreased cardiac metabolism.

Due to the significant role of miR-146a in PPCM pathogenesis, different studies have been conducted on the serum level of miR-146a. Haghikia et al. demonstrated the increased serum levels of Cathepsin D, an enzyme that generates 16 kDa prolactin, miR-146a, N-terminal-pro-brain-natriuretic peptide, and asymmetric dimethylarginine emerged as biomarkers for PPCM [55]. This highly specific biomarker profile can help clinicians detect early-stage PPCM patients and provide appropriate treatment. Overall, the above studies showed that miR-146 physiologically protects the heart against pregnancy overload, but its excessive increase, with a severe decrease in cardiac metabolism, leads to PPCM in high-risk individuals. Therefore, with advances in studies and confirmation of the role of 16 kDa prolactin in diagnosing PPCM, it can be hoped that PPCM can be managed in the early stages by periodical measurement of this factor in high-risk mothers.

Various studies show the therapeutic potential of miR-146A in cardiomyopathies caused by different etiologies, such as DOX-induced cardiomyopathy and DC.

**Figure 3:**
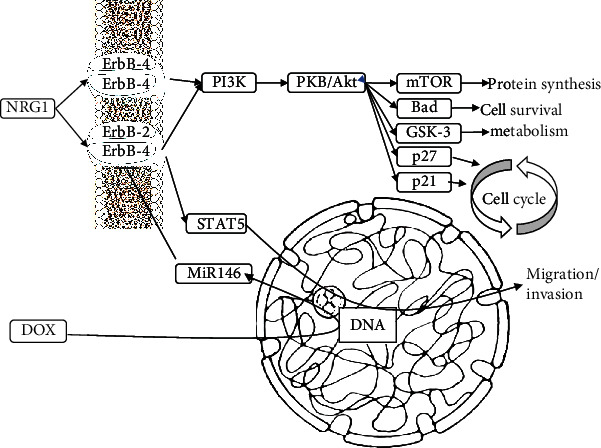
DOX increases the production of miR-146, which reduces cancer cell migration and adhesion. However, higher doses of DOX cause complete inhibition of ErbB-4, affect the PI3K pathway in heart cells, and cause cardiomyopathy by affecting the cell cycle and metabolism. Therefore, developing new therapeutic strategies, such as delivering nucleotides that inhibit miR-146a, can be promising for treating doxorubicin-induced cardiomyopathy.

Doxorubicin (DOX) is an effective anticancer agent. However, its dose-dependent cardiotoxic effects have limited its use. Studies have shown a significant reduction in ErbB4 expression after DOX treatment [56]. Neuregulin-1-ErbB signaling is an essential pathway for normal cardiac function in adults. The inhibition of this pathway by DOX can cause changes in myocardial structure, which can develop into severe and irreversible cardiomyopathy [57]. As shown in Figure 3, the overexpression of miR-146 by DOX can lead to ErbB4 inhibition, which may cause structural problems in myocardiocytes. Therefore, developing new therapeutic strategies based on NRG-1, such as the delivery of nucleotides that inhibit miR-146a, can be promising for treating doxorubicin-induced cardiomyopathy [58].

Considering the role of miR-146 in DC pathogenesis, several studies have focused on the therapeutic capabilities of this microRNA. Randomized clinical trials have shown that even with intensive glycemic control, cardiac complications of T2D cannot be prevented entirely. Costantino et al.'s results indicate that hyperglycemia triggered maladaptive signatures in cardiomyocytes, a phenomenon known as metabolic memory [59], which persisted even after restoring glucose to normal levels. One of the dysregulated miRNAs is miR-146a [60]. This means that miR-146 could be a new therapeutic target in preventing DC. Despite these data, determining this microRNA's exact function in T2D and thus defining treatment strategies based on it requires extensive cell-molecular studies considering the role of this molecule in cardiomyocytes, endothelial cells, and PBMCs.

## 5. Atherosclerosis

Atherosclerosis is the main reason for morbidity and mortality in the Western world. It can cause different complications, such as hypertension, dyslipidemia, and diabetes. Proper management of atherosclerosis can improve the function of endothelial cells and prevent its many complications. For this reason, ACE inhibitors, statins, *β* blockers, and antiplatelet drugs (aspirin, clopidogrel) are often prescribed [61]. Recent studies have suggested new anti-inflammatory approaches for treating atherosclerosis [62].

Different reports suggest that miR-146a could have a significant effect on the reduction of the intracellular cholesterol content of lipid-loaded macrophages [63]. For example, a study reported that miR-146a intravascular injection mimics the protective effects of apoE in both Ldlr−/− and Apoe−/−; Ldlr−/− atherosclerotic mouse models can be considered a novel potential therapeutic strategy [64].

miR-146a produces atheroprotective properties. miR-146a and miR-146b expression are induced in a delayed kinetic manner in ECs by cytokines such as IL-1*β* and TNF-*α*, coinciding with inflammatory gene expression resolution [65]. Cytokine responsiveness in ECs inhibited by overexpression of miR-146a indicates its participation in limiting EC inflammatory signaling by the negative feedback mechanism. Studies showed that the expression of miR-146a is also increased in atherosclerotic plaques in mice and humans [66], directly targeting RNA-binding protein (HuR), and miR-146a repressed both MAPK and NF-*κ*B signaling pathways that inhibit endothelial nitric oxide synthase (eNOS). Also, targeting upstream adaptor proteins IRAK1/2 and TRAF6, the EC adhesion molecules induction is repressed by miR-146a [67]. Contrary to miR-181b, which has a more selective inhibitory role in EC NF-*κ*B signaling pathway, in both macrophages and ECs, the NF-*κ*B signaling pathway is inhibited by miR-146a [68].

To reduce macrophages, ApoE overexpression in ApoE−/− macrophages induces the expression of miR-146a. Also, miR-146a systemic delivery mimics significantly reduce the progression of atherosclerotic lesions. However, along with having an anti-inflammatory role in regulating a range of immune cells (macrophages, T cells, and dendritic cells), miR-146a may also take more part in limiting inflammatory stimuli. Nevertheless, miR-146, another significant cytokine-responsive miRNA in a negative feedback manner, helps in EC inflammation repression [69].

Similarly, in an NF-*κ*B-dependent manner, induction of miR-146a/b in macrophages is involved in resolving inflammation by limiting the cytokine signaling and TLR. It is indicated that apolipoprotein E (apoE), a protein with antiatherosclerotic characteristics, induces the miR-146a expression in macrophages and dumps the inflammatory responses of macrophages in vitro and in vivo. Also, studies suggest that miR-146a may regulate the maturation and secretion of proinflammatory cytokines in dendritic cells by targeting CD40L in ox-LDL-stimulated dendritic cells [68, 70].

In general, due to the anti-inflammatory function of this molecule and various studies, the induction of miR-146 is one of the most promising therapeutic approaches for managing atherosclerotic complications.

Acute coronary syndrome (ACS) is one of the most severe complications of atherosclerosis. In ASC patients, the Th1 activity level is high, which is the consequence of arteriosclerosis activity. Studies have reported that in patients with ACS, the elevation of miR-146a expression level strongly increases the activity of the Th1 cells. Moreover, miR-146a increases proinflammatory factor (TNF-*α*, MCP-1, and NF-*κ*B p65) expression, which plays a vital role in atherosclerosis and ASC progression [71]. Cholesterol crystallization may also be prevented by cholesteryl ester hydrolase inhibition, which converts cholesteryl esters to free cholesterol. In return, ACAT-1 inhibition triggers the promotion of cholesterol crystallization, increased atheroma volume, and major cardiovascular events [72]. Overexpression of miR-146a is considered beneficial in preventing atherosclerosis and its treatment. Although more studies are needed to clarify this issue, miR-146a could be a novel promising regulatory factor in Th1 differentiation and a new therapeutic target for atherosclerosis and ACS [73].

## 6. Heart Failure

Recent studies claim that HF disease can be diagnosed by evaluating the level of miR-146. For example, a study by Beg et al. states that the body begins to produce circulating exosomal miR-146a to counteract the effects of systemic inflammation resulting from HF [74]. As a result, the circulating exosomal miR-146a can be used as a diagnostic biomarker in HF.

Molecular studies have achieved new mechanisms in the pathogenesis of HF. According to most of these studies, miR-146 is considered a critical factor in the pathogenesis of different types of HF. For example, Chouvarine et al. showed that hypoxia could lead to ventricular dysfunction and eventually death by activating the miR-146b-TRAF6-IL-6/CCL2 (MCP-1) pathway [75]; on the other hand, results of research by Heggermont et al. showed that pressure overload leads to decreasing the level of dihydrolipoyl succinyltransferase (DLST) by increasing the level of miR-146a and consequently disrupts the pathway of oxidative metabolism in cardiomyocytes [76]. Oh et al. also showed that this increased level of miR-146a could lead to reduced SUMO1 (small ubiquitin-like modifier 1) expression and SERCA2a (sarcoplasmic reticulum Ca2+-ATPase) SUMOylation in human and animal HF models [77].

Eventually, both studies suggest miR-146a as an essential factor in the course of heart hypertrophy. These results are significant because they consider miR-146a a therapeutic target in HF. Although investigating the association between HF and miR-146a requires further studies, the inhibition of miR-146a may help prevent hypertrophy and HF.

## 7. Future Directions and Potential of miR146 as a Therapeutic Target in CVD

There is a long way before miRNAs can be clinically used in CVD. Research on different miRNAs promised changes in diagnosing and treating these diseases. miR-146 has received much attention, with positive results in most studies. Researches show that this molecule has played a significant role in the pathogenesis of cardiac disease and its related mechanisms. As a result, many potential applications of miR-146 are expected. These potential applications can be examined in three sections: induction, inhibition, and diagnosis.

Secondary to hypoxia and ischemia of a particular tissue, the process of cell apoptosis begins rapidly and leads to irreversible complications. These complications are critical in myocardial tissue, and even with the elimination of hypoxia and the restoration of blood flow to the tissue, the chances of HF and mortality are significantly increased due to apoptosis of cardiac cells [78]. Different studies have been performed in this area, and most of them show that increasing the level of miR-146 (directly or indirectly) leads to a reduction in secondary apoptosis to hypoxia and can somewhat prevent an irreversible complication of MI [79]. With increasing studies and research in this area, the induction of miR-146 may be one of the first approaches to managing MI patients in the future. On the other hand, researches show that miR-146 can have a good effect on myocardial tissue angiogenesis and rapid blood flow to the infarct area by increasing the migration, penetration, and proliferation of coronary artery endothelial cells [80]. This effect of increased angiogenesis is significant in patients with hypercholesterolemia. In these patients, ischemic tissue angiogenesis occurs much less frequently and later than in healthy individuals, making hypercholesterolemia patients more prone to recurrent MI and secondary failure. The research results show that the administration of miR-146b to patients with hypercholesterolemia can accelerate angiogenesis [81]. If future studies confirm these results, miR-146b can significantly reduce the recovery process of heart tissue.

The primary treatment for MI is to increase blood flow with reperfusion methods such as PCI and coronary artery bypass grafting (CABG). Nevertheless, one of the major problems patients face after vascular occlusion and reperfusion is ischemia-reperfusion injury (IRI). Due to ischemia, the tissue is deprived of oxygen and nutrients, resulting in a significant accumulation of inflammatory cytokines. With reperfusion and drainage of retained blood into the bloodstream, cytokines reach other heart parts, causing inflammation and oxidative damage throughout the heart muscle [82].

The treatments to prevent this problem mostly had minimal results and various side effects [83]. One of the treatments is the transplantation of human mesenchymal stem cells (hMSCs). Despite the acceptable results, transplantation of hMSCs may be destroyed by the effects of hypoxia, resulting in treatment failure. Seo et al. show that increasing the level of miR-146 in combination with this treatment significantly increases the survival of hMSCs and, consequently, the efficacy of this method [84]. In contrast, some researchers claim that the self-induction of miR-146 without the help of any additional treatments will protect the myocardium against IRI complications [34].

Another potential therapeutic effect of this miRNA is its protective effect against polymicrobial sepsis. Different research results show that using miR-146 in a preventive way will reduce the cardiac complication of sepsis, such as ICMs [85].

Simultaneously with the widespread use of miRNAs, research has been done on their inhibition, production, and activity. One of the ways to inhibit miRNAs is to use antagomirs. Antagomirs are particular synthetic molecules initially introduced as silencing agents of miRNAs in 2005. One of the unique characteristics of this molecule is that it inhibits a specific miRNA [86]. There are many clinical uses for this selective inhibition. For example, Heggermont et al., by studying the pathogenesis of pressure overload-induced cardiac hypertrophy and its secondary HF, found that it played a crucial role in miR-146 and showed that by inhibiting this miRNA and thus overexpression of its target, dihydrolipoyl succinyl transferase has the ability of heart cells to protect against ventricular dysfunctions [87].

Also, Widmer-Teske et al. indicated that miR-146a acts as a critical regulator of endothelial cell angiogenesis during myocardial regeneration. These results suggest that miR-146a may represent an attractive target for future therapeutic interventions to treat ischemic heart disease [88].

One of the considered applications for miRNAs is their diagnostic usage. This application is specifically for cardiac patients because of the rapid course and severe prognosis of these diseases if not treated in time. Research has shown that the serum level of miR-146 could effectively diagnose MI and prevent LVR or ventricular rupture [38, 40]. miR-146a also reduces inflammation and inhibits complications of sepsis-induced cardiomyopathy by inhibiting the TLR4/NF-*κ*B signaling pathway [43].

Evaluating the molecular level of miR-146 could lead to broader diagnoses in sensitive groups of society. Research has shown that the serum level of miR-146 among patients with diabetic cardiomyopathy and PPCM is higher. Critical needs for diagnosis and earlier treatment of these two diseases can be expected by measuring the periodical level of miRNA of pregnant and cardiac patients. Common cardiomyopathy in these groups will be identified faster and have a better prognosis in treating the patients [54, 60].

Another issue for miR-146 diagnosis is the presence of various polymorphisms of this miRNA. Numerous associations have been reported between polymorphism and the presence or absence of specific cardiac diseases. For example, possibly carriers' mutant type (T allele) of rs2431697 and wild type (C allele) of rs2910164 have a lower risk of developing ACS [25, 28]. Despite the impressive results and the ability of this method to diagnose diseases even before the onset of the first symptoms, the results are highly dependent on the study population; the results may be completely different or even contradictory in other study samples. For this reason, this field will be highly considered in the personal medicine approaches, and a wide range of applications can be expected.

## 8. Conclusion

In conclusion, heart diseases are known as one of the deadliest diseases in most societies, and new research has been able to prevent or treat many of these cases by offering new approaches. In recent years, the discovery of microRNAs has revolutionized the medical sciences, and researchers have since devised various applications for these molecules. Meanwhile, miR-146 has attracted much attention with its essential role in inflammatory processes. Dysregulation of the levels of this microRNA is seen in most heart diseases, but in some of these diseases, such as viral myocarditis or diabetic and prepartum cardiomyopathies, the role of this microRNA is very prominent. In this article, we have tried to gather and review all the studies that point to the correlation between miR-146 and heart disease to provide a more comprehensive view of the progress made and the research paths ahead.

## Figures and Tables

**Table 1:** Descriptive overview of the data extracted from the reviewed studies on the diagnostic application of miR-146a on cardiovascular disease.

Author	Year	Disease	Study design	Participant	Tissue source	Method	MicroRNA	Result	Reference
Oerlemans et al.	2012	ACS	Case-control	332	Serum	QRT-PCR	miR-146a	↑	[16]
Corsten et al.	2010	MI	Case-control	68	Plasma	QRT-PCR	miR-146	—	[20]
Xue et al.	2019	AMI	Case-control	58	Blood	QRT-PCR	miR-146a	↑	[31]
Bukauskas et al.	2019	MI	Case-control	114	Blood	QRT-PCR	miR-146a	↑	[32]
Xie J et al.	2018	CM	In vivo	60	Mice	QRT-PCR	miR-146a	↑	[43]
Besler et al.	2016	CM	In vitro	98	Heart biopsy	QRT-PCR	miR-146b	—	[44]
Gumus et al.	2018	ARM	Case-control	71	Plasma	QRT-PCR	miR-146a-5p	—	[45]
Szczebra et al.	2018	PPCM	Case-control	12	Serum	miRNA cards	miR-146a	↑	[54]
Haghikia et al.	2013	PPCM	Case-control	134	Plasma	QRT-PCR	miR-146a	↑	[55]
Horie et al.	2010	CM	In vivo	N/A	C57BL/6 mice	QRT-PCR	miR-146a	↑	[58]
Beg et al.	2017	HF	Case-control	60	Plasma	QRT-PCR	miR-146a	↑	[74]
Gao et al.	2015	SICD	In vivo	N/A	Mice	QRT-PCR	miR-146a	—	[85]

**Table 2:** Descriptive overview of the data extracted from the reviewed studies on the prognostic application of miR-146a on cardiovascular disease.

Author	Year	Disease	Study design	Participant	Tissue source	Method	MicroRNA	Reference
Arroyo et al.	2018	AF	Case-control	463	Mice, plasma	QRT-PCR/TaqMan analysis	miR-146a	[17]
Chen et al.	2014	MI	Case-control	1808	Blood	PCR-LDR	miR-146a Rs2910164	[24]
Wang et al.	2017	CAD	Case-control	721	Blood	Matrix-assisted laser desorption/ionization time-of-flight mass spectrometry Sequenom Massarray system	miR-146a Rs2431697/Rs2910164	[26]
Zhu et al.	2018	MI	Case-control	774	Blood	PCR-RFLP	miR-146a (C>G)	[27]
Zha et al.	2019	KD	Case-control	246	Blood	PCR-sequence-based typing	miR-146a Rs2910164 G/C, Rs57095329 A/G, Rs6864584 T/C	[28]
Liu et al.	2015	IRI	In vitro	N/A	HUVEC	QRT-PCR	miR-146a/b	[34]
Liu et al.	2015	LVR	Cohort	198	Plasma	QRT-PCR	miR-146a	[38]
Zidar et al.	2011	VR	In vitro	50	Biopsy	QRT-PCR	miR-146a	[40]
Feng et al.	2017	DC	Case-control	N/A	HCMEC, mice	QRT-PCR	miR-146a	[48]
Alipoor et al.	2016	DC	Case-control	375	Blood	PCR-RFLP	miR-146a	[49]
Yang et al.	2011	Ath	In vitro	N/A	Cell line, ATCC	Luciferase assay, Western blot, rescue assay	miR-146a	[63]
Cheng et al.	2013	Ath	In vitro/in vivo	N/A	HUVEC, mice	HUR immunoprecipitation, Western blot, ELISA	miR-146a/b	[65]
Raitoharju et al.	2011	Ath	In vitro	50	Blood, mice	QRT-PCR, Illumina's expression BEADCHIP	miR-146 a/b	[66]
Li et al.	2015	Ath	In vitro/in vivo	N/A	Blood, mice	RT-PCR	miR-146a	[68]

**Table 3:** Descriptive overview of the data extracted from the reviewed studies on the therapeutic application of miR-146a on cardiovascular disease.

Author	Year	Disease	Study design	Participant	Tissue source	Method	MicroRNA	Result	Reference
Palomer et al.	2015	N/A	In vivo	N/A	Mice, AC16 cells	QRT-PCR	miR-146a	—	[14]
Shu et al.	2018	IRI	In vivo	N/A	Rat	QRT-PCR	miR-146a-5p	—	[35]
Zhao L et al.	2019	MI	In vivo	12	Rat	QRT-PCR	miR-146b	↓	[36]
Chen et al.	2015	VM	In vitro	16	Heart biopsy	QRT-PCR	miR-146b	↑	[41]
Halkein J	2013	PPCM	In vivo	N/A	CKO mice	QRT-PCR	miR-146a	↑	[53]
Costantino et al.	2015	DC	In vivo	9	C57/B6 mice	QRT-PCR	miR-146a	↑	[60]
Guo et al.	2010	ACS	In vitro	91	Blood	QRT-PCR,	miR-146a	↓	[71]
Chouvarine et al.	2019	Hypoxia	In vivo	N/A	Rat	QRT-PCR	miR-146b	↓	[75]
Heggermont et al.	2014	HF	In vivo	N/A	Mice	QRT-PCR	miR-146a	—	[76]
Oh et al.	2018	HF	In vivo	N/A	Mice	QRT-PCR	miR-146a	—	[77]
Huang et al.	2016	AMI	In vivo	N/A	Mice	QRT-PCR	miR-146a	—	[79]
Huang et al.	2019	AMI	In vitro	N/A	Endothelial cell	QRT-PCR	miR-146a	—	[80]
Desjarlais et al.	2019	AMI	In vivo	N/A	Mice	QRT-PCR	miR-146b	—	[81]
Seo et al.	2017	IRI	In vitro	N/A	HMSCS	QRT-PCR	miR-146a	—	[84]
Heggermont et al.	2012	Cardiac hypertrophy	In vivo	N/A	Mice	QRT-PCR	miR-146a	—	[87]
Widmer-Teske et al.	2012	AMI	In vitro/in vivo	N/A	Mice, endothelial cell	QRT-PCR	miR-146a	—	[88]

## Data Availability

No data have been submitted to any open-access databases. All data supporting the study are presented in the manuscript or available upon request.

## Abbreviations

CVDs: Cardiovascular diseases
CAD: Coronary artery diseases
MI: Myocardial infarction
ncRNAs: Noncoding RNAs
mRNAs: Messenger RNAs
miRNAs: MicroRNAs
TRAF6: Tumor necrosis factor receptor-associated factor 6
IRAK1: Interleukin 1 receptor-associated kinase 1
TLR: Toll-like receptor
NF-*κ*B: Nuclear factor *κ*-light chain
Tregs: Regulatory T cells
TCR: T-cell receptor
HF: Heart failure
LmiR-146a: Lentivirus expressing miR-146a
STEMI: ST segment elevation myocardial infarction
NSTEMI: Non-ST segment elevation myocardial infarction
ACS: Acute coronary syndrome
AMI: Acute myocardial infarction
ECG: Electrocardiogram
LVR: Left ventricular remodeling
ACE: Angiotensin-converting enzyme
CRP: C-reactive protein
VR: Ventricular rupture
eGFR: Estimated glomerular filtration rate
AF: Atrial fibrillation
VM: Viral myocarditis
ARM: Rheumatic myocarditis
DOX: Doxorubicin
T2D: Type 2 diabetes
DC: Diabetic cardiomyopathy
PBMC: Peripheral blood mononuclear cells
PPCM: Peripartum cardiomyopathy
EC: Endothelial cells
SMC: Smooth muscle cells
eNOS: Endothelial nitric oxide synthase
apoE: Apolipoprotein E
DLST: Dihydrolipoyl succinyl transferase
CABG: Coronary artery bypass grafting
hMSCs: Human mesenchymal stem cells
SUMO1: Small ubiquitin-like modifier 1
SERCa2A: Sarcoplasmic reticulum Ca2+-ATPase
MCP-1: Monocyte chemoattractant protein-1
TNF-*α*: Tumor necrosis factor alpha
KD: Kawasaki disease
IRI: Ischemia-reperfusion injury
SICM: Sepsis-induced cardiomyopathy
Ath: Atherosclerosis
